# Risk factors for oral methotrexate failure in patients with inflammatory polyarthritis: results from a UK prospective cohort study

**DOI:** 10.1186/s13075-018-1544-9

**Published:** 2018-03-20

**Authors:** James Bluett, Jamie C. Sergeant, Alex J. MacGregor, Jacqueline R. Chipping, Tarnya Marshall, Deborah P. M. Symmons, Suzanne M. M. Verstappen

**Affiliations:** 1 Arthritis Research UK Centre for Genetics and Genomics, Centre for Musculoskeletal Research, Manchester Academic Health Science Centre, The University of Manchester, Manchester, UK; 2 Arthritis Research UK Centre for Epidemiology, Centre for Musculoskeletal Research, Institute of Inflammation and Repair, Manchester Academic Health Science Centre, The University of Manchester, Manchester, UK; 3 NIHR Manchester Musculoskeletal Biomedical Research Unit, Central Manchester University Hospitals NHS Foundation Trust, Manchester, UK; 4 Department of Rheumatology, Norfolk and Norwich University Hospital NHS Trust, Norwich, UK

**Keywords:** Methotrexate, Medication persistence, Competing risk analysis, Arthritis, Treatment failure

## Abstract

**Background:**

Oral methotrexate (MTX) is the first-line therapy for patients with rheumatoid arthritis (RA). However, approximately one quarter of patients discontinue MTX within 12 months. MTX failure, defined as MTX cessation or the addition of another anti-rheumatic drug, is usually due adverse event(s) and/or inefficacy. The aims of this study were to evaluate the rate and predictors of oral MTX failure.

**Methods:**

Subjects were recruited from the Norfolk Arthritis Register (NOAR), a primary care-based inception cohort of patients with early inflammatory polyarthritis (IP). Subjects were eligible if they commenced MTX as their first DMARD and were recruited between 2000 and 2008. Patient-reported reasons for MTX failure were recorded and categorised as adverse event, inefficacy or other. The addition of a second DMARD during the study period was categorised as failure due to inefficacy. Cox proportional hazards regression models were used to assess potential predictors of MTX failure, accounting for competing risks.

**Results:**

A total of 431 patients were eligible. The probability of patients remaining on MTX at 2 years was 82%. Competing risk analysis revealed that earlier MTX failure due to inefficacy was associated with rheumatoid factor (RF) positivity, younger age at symptom onset and higher baseline disease activity (DAS-28). MTX cessation due to an adverse event was less likely in the RF-positive cohort.

**Conclusions:**

RF-positive inflammatory polyarthritis patients who are younger with higher baseline disease activity have an increased risk of MTX failure due to inefficacy. Such patients may require combination therapy as a first-line treatment.

**Electronic supplementary material:**

The online version of this article (10.1186/s13075-018-1544-9) contains supplementary material, which is available to authorized users.

## Background

Methotrexate (MTX) is the conventional synthetic disease-modifying anti-rheumatic drug (csDMARD) of first choice for patients with rheumatoid arthritis (RA) [1, 2]. However, response to MTX is not universal; time on ineffective medication may lead to ongoing joint destruction conversely not every patient requires combination DMARD therapy as a first-line treatment [3]. The majority of patients stop MTX due to inefficacy or adverse events; knowledge of predictors of earlier MTX failure may inform treatment pathways in the future.

Female gender, shared epitope positivity, smoking and longer disease duration have previously been associated with MTX failure due to inefficacy [4, 5]. Studies investigating baseline disease activity as a predictor of MTX failure, as measured by change in clinical activity scores over time, have shown conflicting results, suggesting that baseline high disease activity may not be a strong predictor of MTX failure [6, 7]. Poor baseline functional status, as measured by the Health Assessment Questionnaire (HAQ), and female gender are weakly predictive of adverse events [5]. The majority of studies investigating MTX failure commenced recruitment pre-2000 and since this time use of MTX has increased so that it is now the most commonly prescribed csDMARD for RA [8, 9]. There is therefore a need for up-to-date studies that investigate predictors of MTX failure using “real-life” clinical data. Most previous studies have not controlled for competing risks in their analyses. For example, traditional survival analysis may introduce bias by assuming that individuals who have stopped MTX due to an adverse event are still at risk of experiencing MTX failure due to inefficacy [10].

The aims of this study were to (i) investigate the survival of MTX (time to MTX cessation) in patients with inflammatory polyarthritis (IP) in a DMARD-naïve primary care-based inception cohort; (ii) examine patient-reported reasons for stopping MTX in patients with IP recruited after 2000; and (iii) investigate which factors predict MTX failure in these patients utilising competing risks analysis.

## Methods

### Subjects

Subjects were recruited to the Norfolk Arthritis Register (NOAR), a primary care-based inception cohort of patients in the United Kingdom with early inflammatory polyarthritis (IP) [11]. Patients recruited to NOAR between 1 January 2000 and 31 December 2008 who commenced MTX as their first DMARD within 3 months of their baseline visit were eligible for inclusion. Patients were started on a standard dose of MTX (7.5–10 mg/week) and monitored in accordance with UK national guidelines, with dose escalation to a clinically effective dose (up to 25 mg/week). Should MTX be ineffective, a second csDMARD may be offered. The study was approved by the local research ethics committee (REC:15/EE/0076) and all participants provided written informed consent.

### Assessments

Baseline and annual follow-up assessments (1, 2, 3, 5, 7 and 10 years) were conducted by NOAR research nurses. Clinical and demographic data recorded include age at symptom onset, gender, symptom duration, height and weight to calculate body mass index (BMI), baseline smoking status (never smoked, ex-smoker and current smoker), 51 swollen and tender joint count. All patients completed the British version of the HAQ [12]. Patients with at least 1-year follow-up data available were included in this study.

Baseline blood samples were collected and frozen for later analysis. C-reactive protein (CRP) concentration was determined using a Hitachi 917/911 automated analyser (BMG Labtech Ltd, Aylesbury, UK, mg/l). Shared epitope status was tested using the Dynal RELI SSO HLA-DRB1 Typing kit (Dynal, Bromborough, UK). Rheumatoid factor (RF) was measured using a particle enhanced immunoturbidimetric assay where > 40 iU/ml was considered positive (Orion Diagnostica, BMG Labtech Ltd, Aylesbury, UK). Anti-citrullinated protein antibodies (ACPA), as defined by anti-CCP2, were measured using the Axis-Shield DIASTAT kit (Axis-Shield, Dundee, UK) where > 5 U/ml was considered positive. DAS28-CRP(3) was calculated.

### Outcome measures

The primary outcome measure was MTX failure due to adverse events or inefficacy. MTX failure due to adverse events was defined by MTX cessation due to a patient-reported adverse event. MTX failure due to inefficacy was defined by either MTX cessation due to inefficacy, including switching to another DMARD, or the addition of a second DMARD during the study follow-up period. The secondary outcome measure was time to MTX cessation (stopping MTX) for any reason.

Medication details including start and stop date of any DMARD were recorded at each follow-up and patient-reported reasons for cessation of DMARDs were recorded and categorised into stopping due to (i) adverse events, (ii) inefficacy or (iii) other. Adverse event data were categorised according to organ affected (e.g. renal). If patients recommenced MTX treatment within 3 months after MTX cessation, MTX treatment was considered to be ongoing. Patients were followed from recruitment date until MTX failure because of inefficacy, MTX cessation due to an adverse event(s), or last follow-up assessment prior to 18 March 2013. Patients were censored if they stopped MTX for other reasons.

### Statistical analysis

Patient demographics and disease characteristics were summarised using descriptive statistics. Time to MTX cessation was assessed by Kaplan-Meier analysis. The survival probability of patients remaining on MTX treatment, regardless of the addition of a second DMARD, at 2 and 5 years was summarised.

Missing data were imputed using chained equations with transformation of non-normal data, with 20 imputed datasets used. Univariate and multivariate Cox proportional hazards regression models were used to assess potential predictors of MTX failure. Any variable with *p* ≤ 0.2 in univariate analysis was included in the multivariate analysis. Where variables that are highly correlated were significantly associated with MTX failure, the variable with the most missing data prior to imputation was eliminated. Competing risks analysis was undertaken to control for the competing outcome using the Fine-Gray model [13]. A subgroup analysis of those patients fulfilling 2010 EULAR/ACR RA classification criteria was undertaken. All data were analysed using Stata software, version 13 (StataCorp LP, College Station, TX, USA).

## Results

Between 2000 and 2008, 1515 patients were recruited to NOAR and 431 patients were eligible for study inclusion (Additional file 1: Figure S1), patients were followed up for a total of 1608 patient-years. The majority of patients were female and 69% fulfilled the 2010 American College of Rheumatology/European League Against Rheumatism (ACR/EULAR) RA criteria at baseline (Additional file 2: Table S1). Over the study period there were 67 (16%) MTX failures due to adverse events and 143 (33%) due to inefficacy. The most frequent adverse event was gut symptoms, reported by 28 (42%) patients stopping MTX due to an adverse event (Additional file 3: Table S2). The median time to a second-line DMARD being added was 514 days after recruitment (IQR 274–1037).

**Fig. 1:**
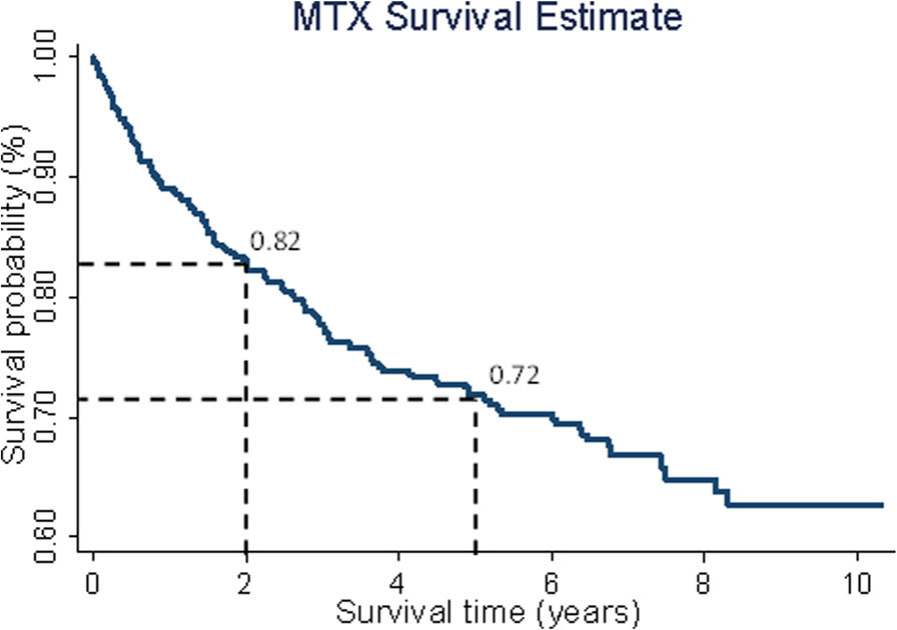
Kaplan-Meier survival estimates for MTX cessation

The probability of patients remaining on MTX at 2 and 5 years was 82% (95% confidence interval [CI]: 0.79–0.86) and 72% (95% CI: 0.67–0.76), respectively. Kaplan-Meier survival curves of time to cessation of MTX therapy are presented in Fig. 1. Subgroup analysis of those who fulfilled ACR/EULAR RA classification criteria revealed a higher probability of remaining on MTX at 2 and 5 years (85% and 77% respectively).

Results of the multivariate Cox and competing risks analysis are presented in Table 1. In the final multivariate models, ACPA status was excluded due to its strong correlation with RF. After adjusting for competing risks, being RF-positive was protective against earlier MTX failure due to an adverse event, being independently associated with later MTX failure (subdistribution hazard ratio [sHR] = 0.34; 95% CI: 0.20–0.59). Younger age (sHR = 0.97; 95% CI: 0.96–0.99), being RF-positive (sHR = 1.67; 95% CI: 1.13–2.48) and higher baseline DAS28-(CRP) (sHR = 1.23; 95% CI: 1.05–1.43) remained associated with earlier MTX failure due to inefficacy (Table 1). Baseline clinico-demographic variables according to reason for MTX failure are presented as Additional file 4: Table S3. Subgroup analysis of those fulfilling 2010 ACR/EULAR RA classification criteria revealed associations trended towards the results for the complete IP cohort, probably due to smaller sample size (Additional file 5: Table S4).

**Table 1:** Multivariate analysis of MTX failure

Variable	Multivariate Cox analysis^a^		Multivariate competing risks analysis^b^	
	Reason for MTX failure		Reason for MTX failure	
	Adverse event (N = 67)	Inefficacy (N = 143)	Adverse event (N = 67)	Inefficacy (N = 143)
Age of disease onset	NR	0.97 (0.96, 0.99) *p* < **0.001**	NR	0.97 (0.96, 0.99) *p* < **0.001**
Female gender	1.39 (0.82, 2.35) *p* = 0.221	1.34 (0.90, 1.99) *p* = 0.143	1.28 (0.78, 2.14) *p* = 0.325	1.23 (0.83, 1.40) *p* = 0.298
BMI	NR	1.02 (0.99, 1.05) *p* = 0.182	NR	1.02 (0.99, 1.05) *p* = 0.184
Current smoker	NR	1.41 (0.86, 2.32) *p* = 0.176	NR	1.43 (0.88, 2.36) *p* = 0.150
Symptom duration	NR	1.00 (1.00, 1.00) *p* = 0.174	NR	1.00 (1.00, 1.00) *p* = 0.120
HAQ score at baseline	1.39 (0.98, 1.96) *p* = 0.064	1.15 (0.88, 1.49) *p* = 0.314	1.34 (0.96, 1.86) *p* = 0.092	1.07 (0.82, 1.40) *p* = 0.606
DAS-28(CRP) at baseline	NR	1.27 (1.10, 1.47) *p* = **0.001**	NR	1.23 (1.05, 1.43) *p* = **0.008**
Shared epitope homozygosity	NR	1.44 (0.81, 2.58) *p* = 0.214	NR	1.42 (0.79, 2.56) *p* = 0.235
Rheumatoid factor positivity	0.37 (0.21, 0.64) *p* < **0.001**	1.44 (0.98, 2.13) *p* = 0.064	0.34 (0.20, 0.59) *p* < **0.001**	1.67 (1.13, 2.48) *p* = **0.011**
ACPA positivity^c^	NR	NR	NR	NR

*NR* not reported, *BMI* body mass index, *HAQ*, Health Assessment Questionnaire, DAS-28(CRP) disease activity score-28-C-reactive protein, *ACPA* anti-citrullinated protein antibody

^a^Values are HR (95% CI)

^b^Values are exponentiated coefficients (exp(beta); subdistribution hazard ratio (sHR)). (95% CI)

^c^Included in univariate model only. Bold values represent *P* < 0.05

## Discussion

Within the past decade there has been a major shift in the treatment paradigm of RA with the aim of achieving low disease activity or remission [2]. This “treat to target” approach incorporates early escalation of MTX dose and use of combination therapy. Knowledge of predictors of MTX failure should enable early identification of individuals who are at increased risk of MTX inefficacy or adverse events and appropriate adjustment in treatment.

Our cohort of patients with IP had a 2- and 5-year probability of remaining on MTX of 82% and 72%, respectively. For those who satisfied the 2010 ACR/EULAR RA classification criteria the 2- and 5-year probability of remaining on MTX was higher at 85% and 77%, respectively. This is higher compared to previous studies of oral and subcutaneous (SC) MTX [8, 14, 15]. Mata et al. reported a 5-year MTX survival of 45% in a cohort of 152 Spanish patients with RA. However, their cohort was recruited pre-2000 and included patients who had failed previous DMARD therapy. Edwards et al. studied participants from the UK General Practice Research Database prescribed MTX for any indication and reported a 57.1% 5-year probability of MTX persistence. Müller et al. investigated the persistence to SC MTX in patients who were DMARD- naïve and demonstrated much lower rates of persistence with only 53% remaining on SC MTX monotherapy at 2 years. The higher MTX persistence seen in the NOAR cohort may therefore be due to a more homogenous cohort of patients starting oral MTX as their first DMARD early within their disease onset. Toxicity remains a concern for patients commencing MTX therapy and high levels of concern are associated with reduced adherence to MTX [16]. In our cohort, 16% of participants stopped MTX due to an adverse event. This is in concordance with previous results reported in the literature [17, 18]. Twenty per cent of patients stopped MTX because of a reported adverse event which could have been identified by routine blood test monitoring. Gastrointestinal side effects were the most frequent self-reported cause of MTX failure due to an adverse event.

In our final model, younger age was associated with earlier MTX failure due to inefficacy. Previous studies investigating baseline age as a predictor of MTX response have found conflicting results [5, 19]. In the SWEFOT trial, younger age was associated with a worse response to MTX in patients with new-onset RA [19]. It is not clear whether younger patients are genuinely less responsive to MTX or whether they are more likely than older patients to move onto combination DMARD therapy in order to achieve complete control of disease activity. Older age *per se* is not a contraindication to earlier combination therapy and older age was not associated with MTX failure due to development of an adverse event in this cohort. Patients started a second-line therapy and were classified as experiencing MTX failure due to inefficacy after a median of 514 days, indicating that combination therapy was not the initial management plan in this cohort and that a second DMARD was initiated due to inefficacy of MTX monotherapy. High disease activity at baseline was associated with early MTX failure due to inefficacy in our cohort as has been reported previously [4]. In contrast to a number of previous studies [5, 6, 19], we found that RF positivity was independently associated with earlier MTX failure due to inefficacy after accounting for the competing risk. Such patients may require consideration of combination therapy as first-line, but further research to evaluate the clinical effectiveness of combination therapy in patients with higher disease activity is required. Varatharajan et al. demonstrated that patients with seronegative arthritis were more likely to cease MTX therapy for all causes [20]. The association between RF negativity and earlier cessation of therapy due to development of an adverse event has not previously been described. One possible explanation for this observation is that patients who are seropositive and have higher disease activity may be more likely to persist with MTX despite gastrointestinal side effects compared to the seronegative cohort with lower disease activity.

Several studies have shown that female gender is associated with earlier MTX failure due to inefficacy [4]. In the present study, female gender was associated with earlier MTX failure due to inefficacy in the univariate model and trended towards earlier MTX failure in the multivariate model. There are several potential explanations for this. Hormonal factors may influence the metabolism of MTX affecting response [5], differences in pain processing between men and women may increase the tender joint count in women [21] or women have a higher baseline disease activity and so are less likely to respond. Current smokers have previously been shown to have a worse response to MTX treatment in early RA. In our data current smoking was associated with MTX failure due to inefficacy in the univariate model only [4]. Smoking may affect MTX metabolism, reducing response and clinicians should therefore support RA patients in smoking cessation in order to maximise response to MTX therapy [22].

## Conclusions

In conclusion, MTX cessation in early inflammatory polyarthritis patients is lower now than in cohorts recruited pre-2000. The data supports ongoing blood monitoring for patients on MTX. Patients at higher risk of MTX inefficacy are those who are younger, RF positive and have higher disease activity at baseline. Such patients may require combination therapy as a first-line treatment.

## Additional files

Additional file 1: **Figure S1.** Flow chart showing recruitment of study participants. (BMP 478 kb)

Additional file 2: **Table S1.** Baseline and follow-up characteristics of the cohort. (DOCX 14 kb)

Additional file 3: **Table S2.** Adverse events leading to MTX failure. (DOCX 13 kb)

Additional file 4: **Table S3.** Baseline clinico-demographic variables according to reason for MTX failure (DOCX 15 kb)

Additional file 5: **Table S4.** Adverse events leading to MTX failure for the subgroup that fulfils 2010 ACR/EULAR RA classification criteria. (DOCX 16 kb)

## Abbreviations

ACPA: Anti-citrullinated protein antibody
ACR/EULAR: American College of Rheumatology/European League Against Rheumatism
BMI: Body mass index
CI: Confidence interval
CRP: C-reactive protein
Cs: Conventional synthetic
DAS-28: Disease activity score-28
DMARD: Disease modifying anti-rheumatic drug
HAQ: Health Assessment Questionnaire
HR: Hazard ratio
IP: Inflammatory polyarthritis
MTX: Methotrexate
NOAR: Norfolk Arthritis Register
NR: Not reported
RA: Rheumatoid arthritis
RF: Rheumatoid factor
SC: Subcutaneous
