# Supplementary figures and images for: Risk factors for oral methotrexate failure in patients with inflammatory polyarthritis: results from a UK prospective cohort study
Source: Arthritis Res Ther. 2018 Mar 20;20:50. doi: 10.1186/s13075-018-1544-9 (PMC5859656; doi:10.1186/s13075-018-1544-9)

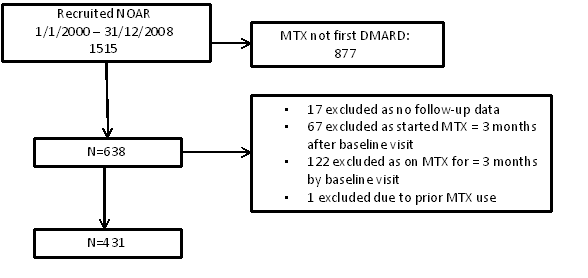

Supplement: Supplementary file 1 — Figure S1. Flow chart showing recruitment of study participants. (BMP 478 kb) [file 13075_2018_1544_MOESM1_ESM.bmp]
