# Supplementary material for: Risk factors for oral methotrexate failure in patients with inflammatory polyarthritis: results from a UK prospective cohort study
Source: Arthritis Res Ther. 2018 Mar 20;20:50. doi: 10.1186/s13075-018-1544-9 (PMC5859656; doi:10.1186/s13075-018-1544-9)
Supplement: Supplementary file 2 — Table S1. Baseline and follow-up characteristics of the cohort. (DOCX 14 kb) [file 13075_2018_1544_MOESM2_ESM.docx]

Table S1. Baseline and follow-up characteristics of the cohort.

| **Baseline Characteristics** |  | **n** |
| --- | --- | --- |
| Age, years | 58 (IQR: 48 - 68) | 431 |
| Female gender, N (%) | 272 (63) | 431 |
| BMI, kg/M^2^ | 26 (IQR: 23 - 30) | 423 |
| Symptom duration, months | 7 (IQR: 4 - 12) | 431 |
| Current smoker, N (%) | 105 (24) | 383 |
| HAQ | 1 (IQR: 0.5 - 1.625) | 429 |
| DAS-28(CRP) | 3.82 (IQR: 3.05 - 4.82) | 381 |
| Shared epitope positive, N (%) | 52 (20) | 255 |
| RF positive, N (%) | 200 (49) | 408 |
| ACPA positive, N (%) | 134 (41) | 330 |
| 2010 ACR/EULAR criteria for RA, N (%) | 297 (69) | 431 |

BMI: body mass index; HAQ: health assessment questionnaire; DAS: disease activity score; RF: rheumatoid factor; ACPA: anti-citrullinated protein antibody; ACR: American College of Rheumatology; EULAR: European Union League Against Rheumatism. Values are mean (SD) or median (IQR).
