# Supplementary material for: Risk factors for oral methotrexate failure in patients with inflammatory polyarthritis: results from a UK prospective cohort study
Source: Arthritis Res Ther. 2018 Mar 20;20:50. doi: 10.1186/s13075-018-1544-9 (PMC5859656; doi:10.1186/s13075-018-1544-9)
Supplement: Supplementary file 3 — Table S2. Adverse events leading to MTX failure. (DOCX 13 kb) [file 13075_2018_1544_MOESM3_ESM.docx]

Table S2. Adverse events leading to MTX failure.

| **Adverse events leading to MTX failure** | **Frequency (%)** |
| --- | --- |
| Gut | 37 |
| Other | 33 |
| Blood | 10 |
| Blood and gut | 4 |
| Missing/Unknown | 4 |
| Skin | 3 |
| Abnormal liver function tests | 3 |
| Renal | 1 |
| Skin and abnormal liver function tests | 1 |
| Skin and blood | 1 |
