# Supplementary material for: Risk factors for oral methotrexate failure in patients with inflammatory polyarthritis: results from a UK prospective cohort study
Source: Arthritis Res Ther. 2018 Mar 20;20:50. doi: 10.1186/s13075-018-1544-9 (PMC5859656; doi:10.1186/s13075-018-1544-9)
Supplement: Supplementary file 4 — Table S3. Baseline clinico-demographic variables according to reason for MTX failure (DOCX 15 kb) [file 13075_2018_1544_MOESM4_ESM.docx]

| **Variable** | **Reason for MTX failure** | | |
| --- | --- | --- | --- |
|  | **All causes (n=245)** | **Adverse event (N=67)** | **Inefficacy (N=143)** |
| Age of disease onset, yrs | 53 (IQR: 43-64) | 59 (IQR: 52-71) | 50 (IQR: 42-61) |
| Female gender, N (%) | 171 (70) | 46 (68) | 103 (72) |
| BMI, kg/m^2^ | 27 (IQR: 23-30) | 27 (IQR: 23-30) | 27 (IQR: 24-31) |
| Current smoker, N (%) | 64 (29) | 12 (18) | 44 (31) |
| Symptom duration, months | 6 (IQR: 5-13) | 6 (IQR: 4-12) | 6 (IQR: 5-14) |
| HAQ score at baseline | 1 (IQR: 1-2) | 1 (IQR: 1-2) | 1 (IQR: 1-2) |
| DAS-28(CRP) at baseline | 3.93 (IQR: 3.19-4.95) | 3.72 (IQR: 3.03-4.73) | 4.37 (IQR: 3.39-5.23) |
| Shared epitope homozygosity, N (%) | 30 (12) | 5 (7) | 23 (16) |
| Rheumatoid factor positivity, N (%) | 109 (44) | 17 (25) | 82 (57) |
| ACPA positivity, N (%) | 70 (29) | 14 (21) | 50 (35) |

**Table S3**. Baseline clinico-demographic variables according to reason for MTX failure

BMI: body mass index; HAQ: health assessment questionnaire; DAS: disease activity score; RF: rheumatoid factor; ACPA: anti-citrullinated protein antibody. Values are mean (SD) or median (IQR).
