# Supplementary material for: Risk factors for oral methotrexate failure in patients with inflammatory polyarthritis: results from a UK prospective cohort study
Source: Arthritis Res Ther. 2018 Mar 20;20:50. doi: 10.1186/s13075-018-1544-9 (PMC5859656; doi:10.1186/s13075-018-1544-9)
Supplement: Supplementary file 5 — Table S4. Adverse events leading to MTX failure for the subgroup that fulfils 2010 ACR/EULAR RA classification criteria. (DOCX 16 kb) [file 13075_2018_1544_MOESM5_ESM.docx]

| **Variable (n=297)** | **Multivariate Cox Analysis^a^** | | **Multivariate Competing Risks Analysis^b^** | |
| --- | --- | --- | --- | --- |
|  | **Reason for MTX failure** | | **Reason for MTX failure** | |
|  | **Adverse event (N=39)** | **Inefficacy (N=119)** | **Adverse event (N=39)** | **Inefficacy (N=119)** |
| Age of disease onset | NR | 0.98 (0.96, 0.99), *p*=**0.002** | NR | 0.98 (0.96, 0.99) p=**0.04** |
| Female gender | 2.14 (0.29, 4.87) p=0.068 | 1.25 (0.80, 1.95) *p*=0.322 | 2.03 (0.90, 4.61) p=0.090 | 1.12 (0.70, 1.77) p=0.634 |
| BMI | NR | 1.03 (0.99, 1.06) *p*=0.135 | NR | 1.03 (0.99, 1.06) p=0.119 |
| Current smoker | NR | 1.55 (0.89, 2.70) *p*=0.119 | NR | 1.62 (0.61, 2.75) p=0.075 |
| Symptom duration | NR | 1.00 (1.00, 1.00) *p*=0.208 | NR | 1.00 (1.00, 1.00) p=0.161 |
| HAQ score at baseline | 1.68 (1.05, 2.67) p=0.030 | 1.08 (0.81, 1.44) *p*=0.591 | 1.57 (1.01, 2.41) p=**0.010** | 1.01 (0.76, 1.34) p=0.962 |
| DAS-28(CRP) at baseline | NR | 1.26 (1.05, 1.51), *p*=**0.012** | NR | 1.21 (1.01, 1.45) p=**0.038** |
| Shared epitope homozygosity | NR | 1.43 (0.71, 2.31) *p*=0.315 | NR | 1.40 (0.70, 2.83) p=0.342 |
| Rheumatoid factor positivity | 0.55 (0.29, 1.04) p=0.066 | 1.40 (0.89, 2.20) *p*=0.149 | 0.51 (0.27, 0.79) p=**0.042** | 1.52 (0.97, 2.39) p=0.067 |
| ACPA positivity^c^ | NR | NR | NR | NR |

Table S4. Adverse events leading to MTX failure for the subgroup that fulfil 2010 ACR/EULAR RA classification criteria.

NR: Not reported. BMI: body mass index; ACPA: anti-citrullinated protein antibody. ^a^Values are HR (95% CI). ^b^ Values are exponentiated coefficients (exp(beta); subdistribution hazard ratio (sHR)). ^c^Included in univariate model only.
